# Secoisolariciresinol Diglucoside with Antioxidant Capacity from Flaxseed: A Study on Microwave-Assisted Germination Optimization

**DOI:** 10.3390/foods14152716

**Published:** 2025-08-01

**Authors:** Jinling Hu, Qingyi Zhang, Yaning Li, Qiqi Zhang, Caihua Jia, Fenghong Huang, Qianchun Deng, Cuie Tang

**Affiliations:** 1 Key Laboratory of Environment Correlative Dietology (Ministry of Education), College of Food Science and Technology, Huazhong Agricultural University, Wuhan 430070, China; hujingling@webmail.hzau.edu.cn (J.H.); 13639642637@163.com (Q.Z.); lyn15035508518@outlook.com (Y.L.); qiqizhang@webmail.hzau.edu.cn (Q.Z.); minttytang@mail.hzau.edu.cn (C.T.); 2 Hubei Key Laboratory of Lipid Chemistry and Nutrition, Oil Crops Research Institute, Chinese Academy of Agricultural Sciences, Wuhan 430062, China; dengqianchun@caas.cn

**Keywords:** lignan, flaxseed, microwave, germination, antioxidant activity

## Abstract

Germination and physical field treatments are processing techniques that have been successfully used to change the amount of active ingredients in flaxseed. However, it is unknown if they work synergistically. This study investigated the effect of microwave-assisted germination on the lignan concentration and antioxidant activity of several flaxseed tissue components. Lignans were primarily dispersed in the flaxseed seed coat. Microwave treatment and germination significantly affected the levels of lignans in various flaxseed sections. Flaxseed hulls’ lignan content and antioxidant activity could be increased by microwave treatment (130 W for 14 s) after germination of 0, 48, or 96 h. Flaxseed kernels lignan content and antioxidant activity could be increased by microwave treatment (130 W for 10 s) before germination. Whole flaxseeds could be improved by microwave treatment (130 W for 10 s) after germination for 72 h. The findings provided a theoretical basis for reducing the loss of lignan resources in flaxseed, enhancing its use as a functional food ingredient, and clarifying the targeted utilization of various lignan sources.

## 1. Introduction

Flaxseeds are one of the most significant oil crops in the world. Flaxseed is extensively farmed in China, United States, and Canada [1,2]. Flaxseed is frequently used in food and feed because it is rich in active nutrients, such as dietary fiber, tocopherols, lignans, linoleic and linolenic acids, and tocopherols [3]. Plant lignans, commonly referred to as phytoestrogens [4], are present in a variety of cereal crops and have a number of physiological benefits, including prevention of vascular disorders, anti-cancer properties, and anti-inflammatory properties [5,6,7,8]. Lignans in flaxseed are dominated by secoisolariciresinol diglucoside (SDG) and secoisolarciresinol (SECO), and most researchers typically characterize the lignan content by the content of SDG [9,10]. The term “lignan” specifically refers to SDG found in flaxseed.

Flaxseed is high in lignans, but the lignans content in flax oil is low. This is because flaxseed oil is extracted from whole seeds; lignans in flaxseed exist primarily in the seed coat, while fat is primarily present in the seed kernel [11]. SDG exists mainly in the hulls of flaxseeds [12]. Flaxseed has a small seed, and the hull–kernel combination is tight, making hull–kernel separation difficult. SDG is generally extracted from flaxseed meals after oil extraction or from an enriched hull fraction after dehulling [13,14]. Hull–kernel separation methods are currently divided into two types of dry and wet methods. Typical process of the dry method is flaxseed milling and sieving [15], which is simple but the separation efficiency is not high, while the typical process of the wet method involves crushing flaxseeds after degumming (not grinding), and then separating by hydraulic vortex, which has a high recovery rate. The spatial distribution of fat and lignan tissues differs, resulting in a limited amount of lignan being transferred to flax oil following flaxseed pressing; thus, increasing the amount of lignan in flaxseed and altering its spatial distribution has garnered considerable research attention. Research findings have shown that flaxseed hull–kernel separation can enrich flaxseed hulls with lignan, which makes it easier to extract lignan from flaxseed hulls and enables flaxseed hulls to become an important source of lignan or a raw material directly applied to functional foods. However, the content of lignan in flaxseed kernels is low [16]. Microwave and germination can serve as a way to treat flaxseed. Germination allows lignan to migrate to the oil phase and increases the lignan content in a short period of time [17]. Microwave pretreatment can also increase the lignan content in flaxseed [18]. We hypothesized that microwave pretreatment in synergy with germination could increase the lignan content and antioxidant activity in flaxseed.

The process of germination, an efficient raw material pretreatment, causes the seeds to become more active, undergo intricate internal physiological changes, and regulates metabolic processes due to the activities of associated enzymes. This alters the nutrients in the seeds [19,20,21,22,23]. Wang et al. investigated how germination affected the amount of nutrients in flaxseed, including lignans. The authors discovered that germination significantly increased the content of lignans in multiple forms, significantly upregulated the expression of genes encoding enzymes involved in lignan secondary metabolism biosynthesis, and markedly increased the amount of phenolics and flavonoids during germination, as well as the in vitro antioxidant activity of oxygen radical scavenging [24].

The use of microwave radiation as a novel pretreatment method is becoming increasingly common in several applications, including seed germination. Following germination synergistic microwave treatment, the oil yield of flaxseed was reported to be significantly increased, the initial cyanogenic glycoside content was significantly reduced, and contents of carotenoids, flavonoids, and sterols were moderately enhanced [25]. The lignan content increased within 5 min with increasing microwave time [26]. Little is known about the impact of microwave synergistic germination treatment on the antioxidant activity of flaxseed and the spatial distribution pattern of lignan in flaxseed. Even so, both microwave and germination treatments have the potential to improve the nutritional value of flaxseed and flaxseed oil.

In this study, the synergistic effects of the combined treatments of microwave and germination were elaborated through investigating the influence of microwave conditions (130 W, 0, 6, 8, 10, 12, 14 s) and germination times (0, 24, 48, 72, 96 h) on the spatial distribution of lignan in flaxseed and the variation patterns in antioxidant activity. The findings provided the theoretical framework to guide the precise exploitation of the nutritional value of flaxseed.

## 2. Materials and Methods

### 2.1. Materials

Flaxseeds (*L. usitatissimum L.*, *Ningya 19*) were obtained from Ningxia Junxingfang Food Technology Co., Ltd. (Ningxia, China). SDG (C_32_H_46_O_16_, chromatographic grade, purity ≥ 97%) was purchased from Sigma-Aldrich (St. Louis, MO, USA). Methanol (CH_3_OH, chromatographic grade, purity ≥ 99.8%; analytical grade, purity ≥ 99.5%), glacial acetic acid (C_2_H_4_O_2_, analytical grade, purity ≥ 99.5%; chromatographic grade purity ≥ 99.9%), sodium hydroxide (NaOH, analytical grade, purity ≥ 96%), hydrochloric acid (HCL, analytical grade, purity 36–38%), 2,4,6-Tri (2-pyridyl)-s-triazine (TPTZ, analytical grade, purity 99%), Iron (II) sulfate heptahydrate (FeSO_4_·7H_2_O, analytical grade, purity 99.0–101.0%), Iron (III) chloride hexahydrate (FeCl_3_·6H_2_O, analytical grade, purity 99%), and sodium hypochlorite (NaClO, analytical grade, purity 5%) were provided by Sinopharm Chemical Reagent Co., Ltd. (Shanghai, China). 1,1-diphenyl-2-picrylhydrazyl (DPPH, analytical grade, purity 98%) was purchased from Shanghai Yuanye Biotechnology Co., Ltd. (Shanghai, China).

### 2.2. Sample Preparation

**Figure 1:**
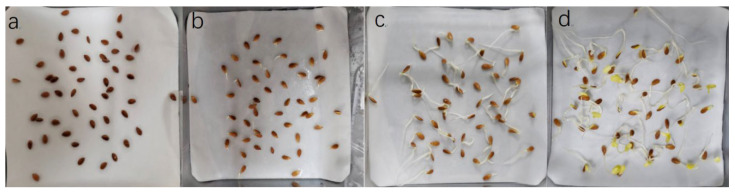
Flaxseeds of different germination periods. 24 h (**a**), 48 h (**b**), 72 h (**c**), 96 h (**d**).

After sterilization with 0.5% sodium hypochlorite solution for 30 s, the flaxseeds were washed three times with ultrapure water. The microwave power for flaxseed treatment was set at 130 W [26]. The treatment durations were set at 6, 8, 10, 12, and 14 s, following the previously established protocol. After microwave pretreatment, the seeds were spread on a germination tray, covered with two layers of medical gauze, and placed in model HP300GS-LED type artificial climate chamber (Wuhan Ruihua Instrument Equipment Co., Ltd., Wuhan, China) at 80% humidity at 24 °C in darkness for 0, 24, 48, 72, and 96 h; flaxseeds at the germination stage are shown in Figure 1. Each sample was freeze-dried in a model (FD-1-50 freeze-dryer, Beijing Bohekang Experimental Instruments Co. Ltd., Beijing, China) and stored at −80 °C.

### 2.3. Extraction, Identification, and Quantitative Determination of Lignans

With a few minor adjustments, a previously described method was used to extract lignans from flaxseeds [27]. About 20 g of freeze-dried flaxseeds were weighed into a grinder and grounded rapidly for 1 to 2 s. The operation was repeated three times. Thereafter, the crushed flaxseeds (not pulverized) were sifted through a sieve until the flaxseed hulls and kernels were weighed separately by about 4 g. The defatted flaxseed (hull and kernel) powder was weighed (1.0000 g, weighed exactly to 0.1 mg). Four milliliters of 80% methanol extract was added, and the sample was processed using a model KQ300DE ultrasonic cleaner (Kunshan Ultrasonic Instruments Co., Ltd., Kunshan, Jiangsu, China) at 300 W for 30 min, followed by 30 min of vortex mixing with a vortex and 20 min of centrifugation 3500× *g* rpm. The supernatant was collected. This extraction was repeated three times and the supernatants were combined for further use.

To prepare a sodium hydroxide (NaOH) concentration of 20 mmol/L in the extraction solution, 0.0064 g of NaOH was added to 8 mL of the extraction solution in a glass tube. The mixture was placed in a water bath at a constant temperature of 50 °C and subjected to alkali-hydrolysis for 12 h. The filtrate was kept in a refrigerator at 4 °C after the alkali solution had been diluted five times and passed through a 0.22 m filter membrane.

The SDG reference standards were precisely prepared as standard solutions corresponding to a concentration range of 0.16 to 1.6 mg of SDG/g of flaxseed. These solutions were analyzed and quantified under established ultra-performance liquid chromatography (UPLC) conditions. A calibration curve was constructed by plotting the concentration of the SDG standards (X, mg of SDG/g of flaxseed) against their corresponding peak areas (Y) using linear regression [27]. Qualitative and quantitative analysis of SDG in the extracts were performed using an ultra-performance liquid chromatography system (Waters Corporation, Milford, MA, USA) equipped with an autosampler and a diode array detector. Chromatographic separation was performed on a BEH Shield RP18 column (100 × 2.1 mm, 1.7 μm) using a gradient of methanol (A) and 0.5% acetic acid in water (B) at 0.2 mL/min. The injection volume was 2 μL with a 35 min run time, and detection was performed at 200–400 nm.

### 2.4. Determination of Antioxidant Activity

#### 2.4.1. Determination of 2,2-Diphenyl-1-picrylhydrazyl (DPPH) Radical Scavenging Activity

Determination of antioxidant activity of different tissue parts of flaxseed by DPPH method was performed with reference to Yu Xiao [28]. DPPH powder (10 mg) was weighed into a beaker, made into a master batch with methanol at a concentration of 2.54 mmol/L, kept at low temperature, shielded from light as a backup, and diluted to 0.0964 mmol/L before use. In a test tube, 2.5 mL of DPPH methanol solution and 0.5 mL of 5-fold diluted lignan extract were combined in a test tube. The test tube was vortexed for 30 s and kept in the light for 30 min. A Multiskan FC fully automated microplate reader (Thermo Fisher Shanghai Instruments Co., Shanghai, China) was used to measure the absorbance at 515 nm after the reaction, with 80% methanol as the blank solution. Aliquots (200 μL) of the reaction solution were added to a 96-well plate. The DPPH radical scavenging rate (DRSR) was calculated as follows:

$$\mathrm{DRSR}\;(\%)\;=\frac{A_{0}{-A}_{1}}{A_{0}}\times 1$$

where A_0_ represents the absorbance value of the control sample and A_1_ represents the sample.

#### 2.4.2. Determination of Ferric-Reducing Antioxidant Power

The antioxidant activity of flaxseed was assessed by fluorescence recovery after photobleaching (FRAP) in a method that was slightly modified from a previously described protocol [29]. To establish the standard curve, six standard solutions of 1.5, 1.2, 0.9, 0.6, 0.3, and 0.15 mM were obtained by gradually diluting 1.5 mM of FeSO_4_ mother liquor. Wells of a 96-well ELISA plate were filled with 10 μL of the standard solution, 30 μL pure water, and 260 μL fresh FRAP working solution. After 10 min in a warm bath at 37 °C, absorbance was measured at 593 nm. The following equation was created by performing a linear regression between the standard solution Fe^2+^ concentration (X, mM) and the associated absorbance (Y):

Y = 0.7314X − 0.0395, R^2^ = 0.9916

For sample analysis, wells of a 96-well plate were filled with 10 μL of lignan extract solution, 30 μL pure water, and 260 μL freshly prepared FRAP working solution. The plate was incubated for 10 min at 37 °C and the absorbance was measured at 593 nm using a microplate reader. A standard curve equation was used to determine the antioxidant activities of the sample solutions.

### 2.5. Statistical Analysis

The experimental results are expressed as mean ± standard deviation. All experiments were repeated three times. Data analysis and charting were performed using Excel 2016 and SPSS version 23.0, respectively. One-way analysis of variance (ANOVA) and multiple comparison using Duncan’s test were used to test for statistical differences, with the significance level set at *p* < 0.05.

## 3. Results and Discussion

### 3.1. Effect of Microwave Duration and Germination Time on Tissue Spatial Distribution Pattern of Lignan Content in Flaxseeds

#### 3.1.1. Analysis of Lignan Content in Flaxseed Hulls

**Figure 2:**
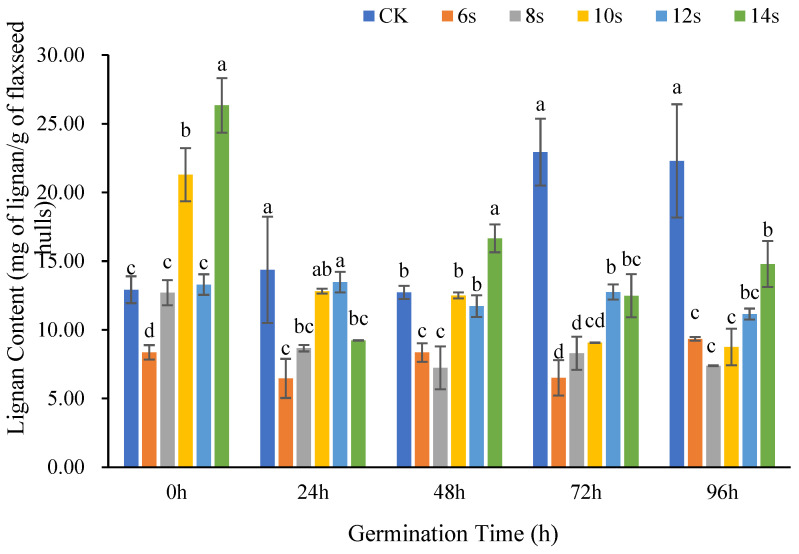
The content of lignan in flaxseed hulls during germination. Values are means ± SD (*n* = 3). Different letters indicate significant differences among microwave treatment groups (*p* < 0.05) by one-way ANOVA followed by Duncan’s test.

Figure 2 showed the changes in lignan content in flaxseed hulls at various germination times following different microwave treatment times. The CK group was not microwaved and was the blank control. At the same germination, the lignan content usually did not increase or decrease monotonically with the microwave time, but showed a tendency of decreasing and then increasing, especially at 0 h, 48 h, and 96 h of germination. The lignan in the treatments with shorter microwave times of 6 s and 8 s were generally lower than those in control without microwave treatment. At the germination of 0 h, 48 h, and 96 h, the maximum lignan content appeared in the microwave 14 s treatment. At the germination of 24 h and 72 h, the maximum appeared in the microwave 12 s treatment. This suggested that a short microwave time may not be sufficient to effectively release lignan, and that a longer microwave time was critical to enhance the lignan content of flaxseed hulls. However, too long a microwave time may cause the material to be charred or even destroy the target product [30]. The specific microwave time threshold requires further investigation.

At the same microwave duration, prolonged germination usually resulted in lower lignan content. In the microwave 14 s treatment, the lignan content was highest at 0 h germination, and steadily decreased by 48 h and 96 h of germination. This suggested that prolonged germination negatively affected lignan content [31]. The main reason may be that the roles of lignan in flaxseeds are antioxidation and biological defense [32]. Lignan content was dynamically affected by both consumption and synthesis during germination. Firstly, when the flaxseeds were subjected to incremental physiological stress, lignan consumption significantly increased to ensure their survival during prolonged germination [33]. Secondly, physical field treatment may also affect the activity of enzymes related to lignan decomposition, promoting lignan consumption [34].

The magnitude of the physical field processing energy input is primarily controlled by microwave time. This energy has no linear impact on the lignan content of the flaxseed hulls. In the present study, the overall pattern of reduction was followed by an increase with shorter microwave durations, but a decrease in the lignan content was observed in the same germination period when different microwave times were used as pretreatments. Under these conditions, it is advantageous to increase the lignan content by appropriately extending the microwave treatment time. The amount of lignan in flaxseed hulls decreased when the germination time was prolonged along with supplementary microwave pretreatment. The pattern was similar when the same microwave time was used for pretreatment with varied germination times.

#### 3.1.2. Analysis of Lignan Content in Flaxseed Kernels

**Figure 3:**
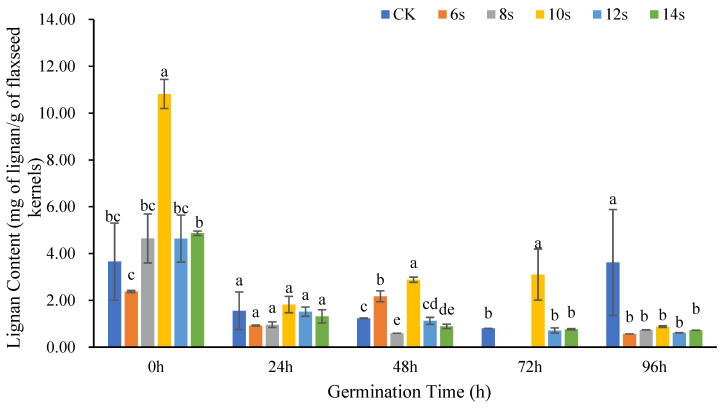
The content of lignan in flaxseed kernels during germination. Values are means ± SD (*n* = 3). Different letters indicate significant differences among microwave treatment groups (*p* < 0.05) by one-way ANOVA followed by Duncan’s test.

Figure 3 showed the changes in lignan content in flaxseed kernels at different germination times after different microwave treatment times. Overall, the lignan content in flaxseed kernels at different germination periods was lower than that in flaxseed hulls at the same germination period. At 0 h of germination, the lignan content of flaxseed kernel in the 10 s microwave treatment reached the maximum, which was significantly higher than that in the control. When the microwave time exceeded 10 s, the lignan content decreased with the extension of microwave time. Except for 0 h of germination, the lignan content in other periods was predominantly lower than that in the control and showed a declining trend with the extension of germination time.

Except for the germination of 0 h, the lignan content in groups with 8 s microwave pretreatment combined with different germination periods was lower than that in the groups without microwave pretreatment at the same germination period, which was consistent with flaxseed hulls. In contrast to flaxseed hulls, the lignan content of the treatment was maximum at the same germination period after a 10 s microwave synergistic pretreatment. However, the 14 s microwave pretreatment, which was most beneficial for increasing the lignan content in flaxseed hulls, did not show advantages for flaxseed kernels. When the microwave duration was increased from 8 to 12 s, the lignan content showed an increasing and then decreasing trend. However, microwave pretreatment of the 14 s was most beneficial for increasing the lignan content in flaxseed hulls, which showed no advantage for flaxseed kernels.

The foregoing findings indicated that the lignan content of flaxseed kernels was much higher under the ungerminated treatment than in the germination treatments. This was not evident with flaxseed hulls. Ungerminated flaxseed hulls were tightly bound to the kernel and lignan is primarily present [35]. This may reflect the lower rate of hull–kernel separation in the ungerminated state, and a higher proportion of kernels mixed with hulls, resulting in a high lignan content in the ungerminated group of flaxseed kernels.

#### 3.1.3. Analysis of Lignan Content of Whole Flaxseeds

**Figure 4:**
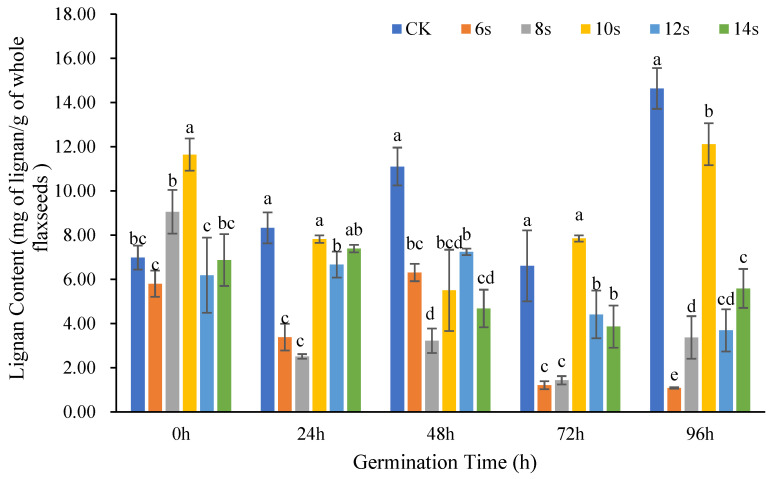
The content of lignan in whole seeds of flaxseed during germination. Values are means ± SD (*n* = 3). Different letters indicate significant differences among microwave treatment groups (*p* < 0.05) by one-way ANOVA followed by Duncan’s test.

The changes in lignan content in whole flax seeds at different germination time periods after treatment with different microwave times are shown in Figure 4. At 0 h, 24 h, 72 h, and 96 h of germination, the treatments all reached the maximum in 10 s microwave pretreatment, yet the lignan content were lower than the control. For the treatment of 72 h germination combined with 10 s microwave, the lignan content was higher than the control.

Whole flaxseeds changed in a similar way to flaxseed hulls and kernels in that their lignan concentration decreased at various germination periods in tandem with 6 or 8 s duration of microwave treatment. The highest lignan concentration in the treatment was achieved after the 10 s microwave treatment. This finding indicated that when the microwave duration was extended from 8 to 10 s, the lignan content in whole flaxseeds increased. Whereas the lignan content decreased when the treatment time prolonged from 10 to 12 s. This pattern was consistent with the change trend of flaxseed kernels.

The analysis of the changes in lignan content in the flaxseed components supported the conclusion that in flaxseed hulls, kernels, and whole seeds, the amount of lignan was reduced when microwave pretreatment for 6 and 8 s is combined with germination. Combined with Yang Ruinan’s research, it was speculated that the possible reason for the increase in lignan content in flax oil within 5 min of microwave pretreatment was the migration of lignans into the oil phase [26].

Suri et al. found that only a small number of phenolic acids and flavonoids in flaxseed migrated into the oil phase during cold press extraction, and this migration was improved by thermal pretreatment [36]. Microwaving for 10 s was ideal for increasing lignan content in flaxseed kernels and flax whole seeds at most germination times. However, for flaxseed hulls, the highest lignan level under microwave pretreatment conditions occurred at 12 or 14 s. These findings suggested that the lignan content in flaxseed hulls declined while flaxseed hull lignan content increased when the microwave duration was increased from 8 s to 10 s. It is conceivable that some of the lignan in the flaxseed hulls might move to the flaxseed kernel or oil phase after the 10 s microwave irradiation. Prolonged microwave pretreatment was only advantageous for the enrichment of lignan in flaxseed hulls. This pretreatment was detrimental for kernels as well as whole seeds.

### 3.2. Effects of Microwave Duration and Germination Time on the Spatial Distribution Pattern of Antioxidant Activity in Flaxseed Tissues

#### 3.2.1. Analysis of Antioxidant Activity of Flaxseed Hulls

**Figure 5:**
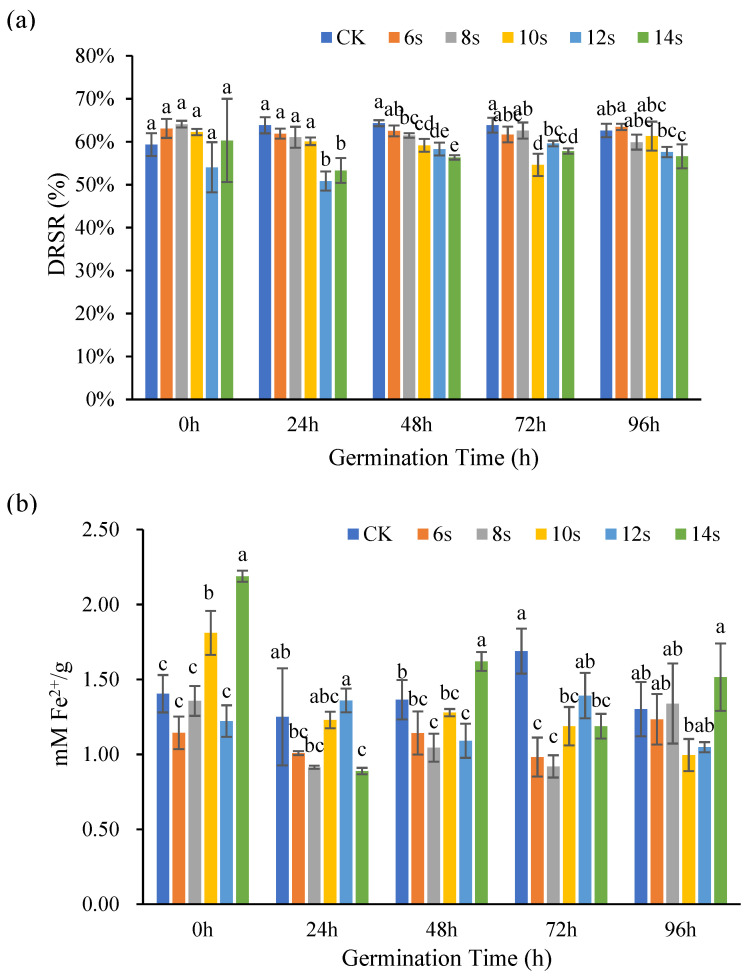
The antioxidant capacity of flaxseed hulls during germination. DPPH (**a**) and FRAP (**b**). Values are means ± SD (*n* = 3). Different letters indicate significant differences among microwave treatment groups (*p* < 0.05) by one-way ANOVA followed by Duncan’s test.

The antioxidant capacity of flaxseed hulls at different germination periods was determined by DPPH method and FRAP method, and the results are shown in Figure 5. In the germination treatments, DRSR decreased with increasing microwave time for the same germination period. The DRSR did not change significantly in the ungerminated control with increasing microwave time. Overall, the synergistic microwave and germination had no impact on the DRSR of flaxseed hulls. The effects of other antioxidant chemicals, such as phenolic acids and flavonoids in flaxseed hulls, could explain why changes in DRSR were not consistent with changes in lignan concentration [37,38].

FRAP results were expressed as mM Fe^2+^/g, with higher numbers indicating greater Fe^2+^ scavenging capacity. During the 0 h germination, the antioxidant capacity of the treatment was up–down–up with the microwave treatment time, and the 10 s and 14 s treatments increased significantly compared to the control. During the 24 h germination, the antioxidant capacity of the treatment increased with the microwave treatment time. Except for the 12 s treatment, the antioxidant capacity was lower than that of the control. During the 48 h germination, the antioxidant capacity of the treatment increased with the extension of the microwave treatment time, and the antioxidant capacity of the 14 s treatment increased significantly compared with that of the control. In the 72 h germination period, with the extension of microwave treatment time, the antioxidant capacity of the treatment first increased and then decreased, the maximum value was obtained in the 12 s treatment, and the antioxidant capacity of the treatment was lower than that of the control. In the 96 h germination period, the antioxidant capacity of the 8 s and 14 s treatments were higher than that of the control. Overall, the antioxidant capacity decreased first and then increased with the extension of microwave time, which was like the change trend of lignan [39]. These experimental findings suggested that lignan is a significant contributor to the antioxidant activity of flaxseed hulls, but the content of lignan also decreased with the extension of germination time, so long-term germination will affect its antioxidant activity through reducing the content of lignan.

#### 3.2.2. Analysis of Antioxidant Activity of Flaxseed Kernels

**Figure 6:**
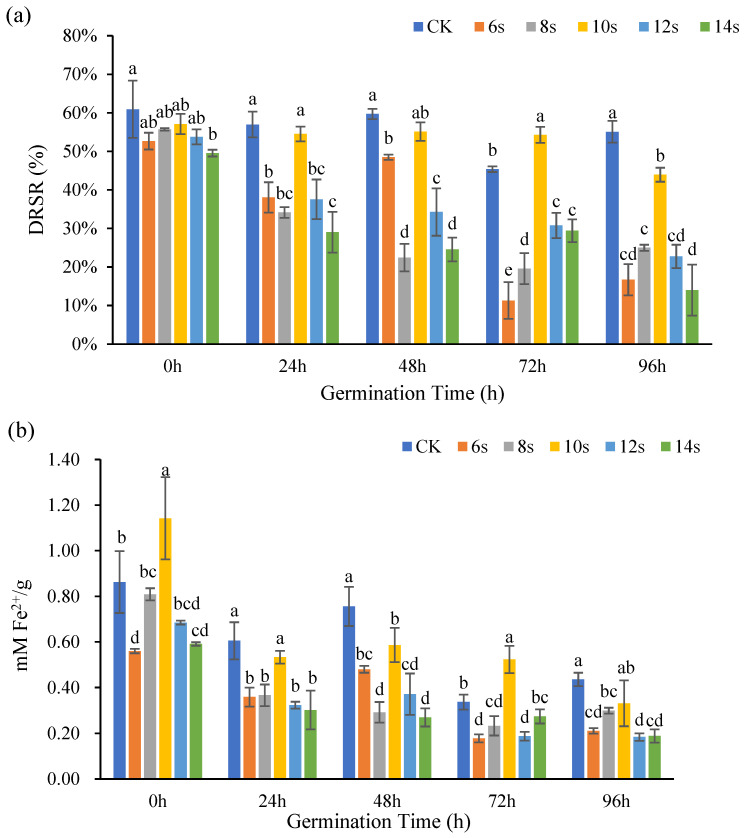
The antioxidant capacity of flaxseed kernels during germination. DPPH (**a**) and FRAP (**b**). Values are means ± SD (*n* = 3). Different letters indicate significant differences among microwave treatment groups (*p* < 0.05) by one-way ANOVA followed by Duncan’s test.

The antioxidant capacity of flaxseed kernels at different germination periods were determined by DPPH method and FRAP method, and the results were shown in Figure 6. At 0, 24, 48, 72, and 96 h of germination, the treatments consistently achieved maximum DRSR values at 10 s, though these values remained lower than those of the control. However, only the 72 h germination treatment group exhibited higher DRSR values compared to the control.

Overall, there was a favorable correlation between the lignan concentration of flaxseed kernels and the ability of the treatments and control to scavenge Fe^2+^ at various germination time points. At 0 h of germination, the antioxidant capacity of treated samples initially increased then decreased with prolonged microwave treatment, reaching its maximum at 10 s. This treatment showed significantly enhanced antioxidant activity compared to the control, indicating the lignan content variation observed simultaneously in flaxseed kernels. For all germination periods (24 h, 48 h, 72 h, and 96 h), the 10 s microwave-assisted groups consistently exhibited peak values. However, a statistically significant increase in Fe^2+^ chelating activity was observed exclusively in the 10 s treatment at 72 h germination when compared to the control. Notably, at 72 h of germination, lignan content in both 6 s and 8 s of microwave treatments were below detectable levels, while antioxidant capacity was still observed in these samples.

Studies have demonstrated that methanolic aqueous extracts of flaxseed also contain phenolic acids and flavonoids in addition to lignan, and that the combination of lignan, phenolic acids, and flavonoids strengthens the antioxidant system of flaxseed [40]. In the present study, lignan was not detected in flaxseed kernel extracted 72 h after germination and 6 s of synergistic microwave treatment, but antioxidant capacity was still measurable. Alternatively, the lignan content was low and the antioxidant activity was high under certain conditions, likely because the flaxseed kernel extract also contained phenolic acids or flavonoids, which had antioxidant activity. Since flaxseed kernels contained more fat and were typically used to extract flax oil, the results also suggested that microwave treatment for 10 s could increase the antioxidant capacity of ungerminated flaxseed kernels. This finding could inform approaches that increase the stability of flax oil.

#### 3.2.3. Analysis of Antioxidant Activity of Flax Whole Seeds

**Figure 7:**
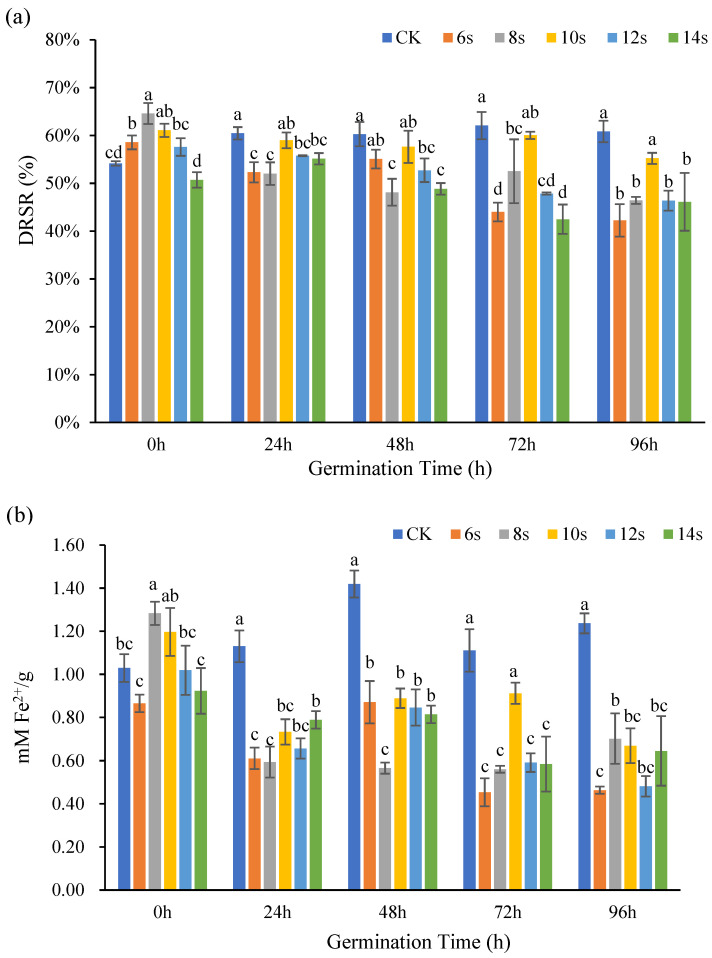
The antioxidant capacity of whole seeds of flaxseed during germination. DPPH (**a**) and FRAP (**b**). Values are means ± SD (*n* = 3). Different letters indicate significant differences among microwave treatment groups (*p* < 0.05) by one-way ANOVA followed by Duncan’s test.

The antioxidant capacity of whole flax seeds at different germination times was determined by DPPH method and FRAP method, and the results were shown in Figure 7. The DRSR of the treatments first increased and subsequently declined throughout the 0 h germination. The DRSR of the 6, 8, and 10 s treatments increased significantly in comparison to the control. The DRSR was highest after 8 s of microwave treatment. The DRSR of the 10 s treatment group was highest 24, 48, 72, and 96 h after germination, with all the values exceeding the value of the control.

During the 0 h germination, the ability of the treatments to remove Fe^2+^ increased from 0.87 ± 0.04 mM/g to 1.28 ± 0.05 mM/g for both the 8 s and 10 s microwave treatments. The antioxidant capacity was greater than that of the control. At 24 h and 48 h of germination, the antioxidant capacity of the treatment decreased–increased–decreased, and the antioxidant capacity of the treatment was lower than that of the control. During the 72 h period of germination, the antioxidant capacity of the treatment was lower than that of the control, and with the extension in microwave time, the antioxidant capacity of the treatment increased first and then decreased. At 24, 48, and 72 h of germination, the antioxidant capacity peaked at 10 s of microwave treatment. At 96 h of germination, with an increasing–decreasing–increasing trend in the treatment, the Fe^2+^ scavenging capacity peaked at 8 s. However, in each of these periods, the antioxidant capacity of the treatment was lower than that of the control. When two antioxidant activity markers were used in combination, microwave treatments of 8 or 10 s demonstrated a more pronounced effect on enhancing antioxidant activity.

Wang et al. [41] investigated the changes in phenolic acid and flavonoid morphology over a 10-day germination time, and the results showed that the free state phenolic acid and flavonoid content gradually increased as the germination time increased, while Huang et al. showed that in free state, the antioxidant capacity of phenolic acids was stronger than that of the bound state [42]. In the present study, microwave treatment significantly influenced the antioxidant capacity of whole flaxseeds during germination. The observed reduction in antioxidant activity may be attributed to microwave-induced alterations in the composition and content of phenolic acids and flavonoids, consequently affecting the overall antioxidant potential of the germinated whole seeds.

## 4. Conclusions

Flaxseed lignan was primarily found in the hulls. The 130 W microwave treatment for 14 s followed by germination of 0, 48, or 96 h can increase the lignan content and antioxidant capacity of flaxseed hulls. The lignan and antioxidant capacity of flaxseed kernels can be greatly increased by 130 W microwave treatment for 10 s followed by germinating for 72 h. The tissue space migratory characteristics of lignan during the germination of flax whole seeds may be affected by pretreatment with physical fields, which may also encourage their migration to the oil phase of the flaxseed kernel. The combination of microwave treatment for 10 s at 130 W with 0 h or 72 h germination had a greater impact on the lignan and antioxidant capabilities of flax whole seeds. Diverse flaxseed sections can be chosen based on their different purposes since the lignan concentration of each part varies depending on the microwave-assisted germination time. For lignan extraction and enrichment, flaxseed hulls subjected to 130 W microwave treatment for 14 s followed by 0, 48, or 96 h germination are recommended. For oil extraction and whole-seed food applications, optimal conditions include 130 W microwave treatment for 10 s with 0 h or 72 h germination.

Microwave synergistic germination could regulate the intricate physiological and biochemical processes involved in flaxseed germination. The amount of lignan in different flaxseed fractions increased, and its antioxidant capacity was improved. The finding provides a theoretical basis for reducing the loss of lignan resources in flaxseed and promoting its comprehensive use as a functional food ingredient, as well as the targeted utilization of various lignan sources.

## Data Availability

The original contributions presented in the study are included in the article, further inquiries can be directed to the corresponding authors.
